# Near-Stasis in the Long-Term Diversification of Mesozoic Tetrapods

**DOI:** 10.1371/journal.pbio.1002359

**Published:** 2016-01-25

**Authors:** Roger B. J. Benson, Richard J. Butler, John Alroy, Philip D. Mannion, Matthew T. Carrano, Graeme T. Lloyd

**Affiliations:** 1 Department of Earth Sciences, University of Oxford, Oxford, United Kingdom; 2 School of Geography, Earth and Environmental Sciences, University of Birmingham, Birmingham, United Kingdom; 3 Department of Biological Sciences, Macquarie University, New South Wales, Australia; 4 Department of Earth Science and Engineering, Imperial College London, London, United Kingdom; 5 Department of Paleobiology, Smithsonian Institution, Washington, D.C., United States of America; University of California, Berkeley, UNITED STATES

## Abstract

How did evolution generate the extraordinary diversity of vertebrates on land? Zero species are known prior to ~380 million years ago, and more than 30,000 are present today. An expansionist model suggests this was achieved by large and unbounded increases, leading to substantially greater diversity in the present than at any time in the geological past. This model contrasts starkly with empirical support for constrained diversification in marine animals, suggesting different macroevolutionary processes on land and in the sea. We quantify patterns of vertebrate standing diversity on land during the Mesozoic–early Paleogene interval, applying sample-standardization to a global fossil dataset containing 27,260 occurrences of 4,898 non-marine tetrapod species. Our results show a highly stable pattern of Mesozoic tetrapod diversity at regional and local levels, underpinned by a weakly positive, but near-zero, long-term net diversification rate over 190 million years. Species diversity of non-flying terrestrial tetrapods less than doubled over this interval, despite the origins of exceptionally diverse extant groups within mammals, squamates, amphibians, and dinosaurs. Therefore, although speciose groups of modern tetrapods have Mesozoic origins, rates of Mesozoic diversification inferred from the fossil record are slow compared to those inferred from molecular phylogenies. If high speciation rates did occur in the Mesozoic, then they seem to have been balanced by extinctions among older clades. An apparent 4-fold expansion of species richness after the Cretaceous/Paleogene (K/Pg) boundary deserves further examination in light of potential taxonomic biases, but is consistent with the hypothesis that global environmental disturbances such as mass extinction events can rapidly adjust limits to diversity by restructuring ecosystems, and suggests that the gradualistic evolutionary diversification of tetrapods was punctuated by brief but dramatic episodes of radiation.

## Abstract 2

A large-scale examination of the fossil record reveals slow diversification rates in tetrapods across the 190-million-year Mesozoic era, followed by a possible rapid and abrupt 4-fold increase in species richness after the Cretaceous-Paleogene mass extinction event.

## Author Summary

Vertebrates invaded the land more than 360 million years ago. Since then, they diversified to more than 30,000 tetrapod species today, including birds, mammals, squamates, and amphibians. The fossil record provides our best window onto diversification across such long spans of time, but is unevenly sampled. Previous studies counted observed families of fossil tetrapods and supported an expansionist model, entailing large and unbounded diversity increases through time. We applied methods that correct for differences in sampling through time and space to a comprehensive species-level database of Mesozoic to early Cenozoic fossil tetrapods. We find strong evidence that tetrapod diversity increased during the Mesozoic, but that the long-term net rate of diversification was low; species richness only doubled or tripled over 190 million years. This is enigmatic because today’s high biodiversity could not have been realised at such a slow rate. Diversification rates must have been much higher during other intervals, or rapid diversification might have been concentrated during brief episodes such as the earliest Cenozoic. Patterns of diversification on geological timescales and their relationships to hypothesised drivers such as ecological opportunity and environmental volatility must receive renewed scrutiny if we are to understand how land vertebrates and other animals attained the high biodiversity seen today.

## Introduction

Tetrapods, the limbed vertebrates, include mammals, amphibians, and reptiles (including birds), and today comprise more than 30,000 species. Alongside plants and insects, they are key components of the non-marine biota and play a diverse range of ecological roles. Patterns of tetrapod diversification from their Late Devonian origin to the present day are therefore central to understanding the evolution of terrestrial ecosystems.

Almost all extant tetrapod species belong to just a few hyper-diverse groups, including neoavian birds, placental mammals, frogs, and squamates (e.g., [1]). Although both fossils and molecular clock analyses indicate Mesozoic origins for these hyper-diverse groups [2–7], there is significant controversy over the timing of major increases in their species diversity. This controversy is embodied by disagreements about the divergence times of Placentalia and Neoaves, the clades that include most extant mammal and bird species. For example, fossils suggest that placental mammals were either absent, or so rare as to be undiscovered, prior to the end of the Cretaceous [8], and phylogenomic studies of birds indicate that deep neoavian divergences were concentrated in the earliest part of the Cenozoic [9]. This evidence contradicts most other molecular clock estimates, which imply substantial origination of higher taxa within Placentalia [5] and substantial species diversification within Neoaves [6] before the Cenozoic. Nevertheless, the timings of deep divergences within extant tetrapod clades have generally been interpreted as supporting an “expansionist” mode of diversification, under which unbounded and essentially exponential diversification led to significant, near-continuous increases in species richness on land, especially since the late Mesozoic [2,10,11].

Patterns of fossil tetrapod diversity have also been interpreted as evidence of expansionist diversification on land [2,10–14]. This contrasts with strong evidence for constrained diversification in the fossil records of shallow marine animals [15–19], planktonic Foraminifera [20,21], North American mammals [22], and mammalian subgroups [23–25]. These groups have rich, densely sampled fossil records that demonstrate the existence of diversity-dependent controls of diversity patterns. Under diversity dependence, speciation rates, extinction rates, or both vary with standing diversity such that net diversification rates approach zero or become negative when diversity is high [15,26–28]. This has the general effect of “flattening” diversity curves and generating long intervals of near-static diversity, but need not imply a permanently fixed upper limit to species richness (e.g., [28]). The population- and community-level processes causing diversity dependence on macroevolutionary scales are not well understood. However, it is possible that the availability of ecological opportunity regulates species richness within local communities via agonistic interspecies interactions such as competition over finite resources, thereby influencing global patterns of diversification through time [15,17,29].

The question of whether expansionist [2,30] or more constrained [15,16,18,31] patterns of diversification characterise the evolution of life on Earth is among the most contentious macroevolutionary questions [10,28,29,32,33]. Its answer has substantial implications for the origins and future of the enormous scope of extant biodiversity (estimated at 2–8 million species [34]), and for assessing whether equilibrial processes inferred from the study of island biogeography are applicable to global spatial scales and geological time spans [26–28,32]. Animal diversity on land is especially high, comprising 75%–95% of multicellular species on Earth [35,36], and evolved in significantly less time than did the lower diversity of marine animals [11]. This observation has been used to justify an “emerging consensus” that species diversification on land was essentially exponential, irrespective of the evidence for constrained diversification in the marine realm [10,11].

The expansionist paradigm implies that ecological constraints on diversification rates are either non-existent or unimportant in determining patterns of global diversification on geological timescales [2,32]. This could be possible if competitive ecological interactions among species are rare, or if their effects are typically weakened by evolved responses such as niche partitioning (e.g., [29,37]). Under an expansionist model, per-lineage net diversification rates in major clades of terrestrial animals have been positive and high on long timescales, commensurate with the attainment of high biodiversity in the present (although other patterns, such as mass extinction events and adaptive radiations, may be evident on shorter timescales) [2,13]. This model implies that substantial relative increases in species richness should occur during time intervals spanning hundreds of millions of years. Nevertheless, patterns of diversity across all tetrapods on land and their implications for macroevolutionary dynamics at larger scales have not been rigorously characterised on geological timescales, so this prediction has not been explicitly tested.

So far, the fossil evidence for strongly expansionist diversification on land is based on iterations of a 30-year-old compendium of the geological ranges of non-marine tetrapod families [12,38]. This range-based, family-level approach has three shortcomings. Firstly, although range-based approaches have some utility in filling the unsampled gaps within fossil taxon ranges, counts of range-based data do not address core biases affecting fossil diversity counts, such as uneven sampling of specimens, environments, or geographic space through geological time, or the “Pull of the Recent” and related effects [19,39–41]. Range-based approaches are also prone to edge effects ([42]; an issue that also applies to phylogenetic diversity estimates [43]). Secondly, the composition of taxonomic families is determined by an inconsistent set of subjective criteria including phenotypic distinctiveness, species diversity, and phylogenetic monophyly (e.g., [44]). We do not know the “perceptual algorithms” governing the delimitation of named clades such as families, or how their application varies among geological intervals or across the Tree of Life. However, we do know that they can profoundly bias studies of diversification [45]. Thirdly, and perhaps most importantly, the processes of evolutionary diversification act directly only on individual evolving lineages and so are most adequately represented at species level.

We analysed non-marine (terrestrial plus freshwater) diversity patterns across Tetrapoda, applying sample standardization approaches [18,46] to species occurrence data from the Paleobiology Database (http://paleobiodb.org), accessed via Fossilworks (http://fossilworks.org) on 22 January 2015. These data result from a concerted effort to document the Mesozoic–early Paleogene (Ypresian) tetrapod fossil record, led by the authors of this paper, and representing an estimated 6,520 h of work by more than 70 contributors [47]. Flying taxa with Lagerstätten-dominated records (birds, bats, pterosaurs) that provide little robust information on species richness were excluded from our analyses, the implications of which are discussed below (see Materials and Methods and Results and Discussion).

## Results and Discussion

### Analytical Results: Observed Diversity Counts

**Fig 1:**
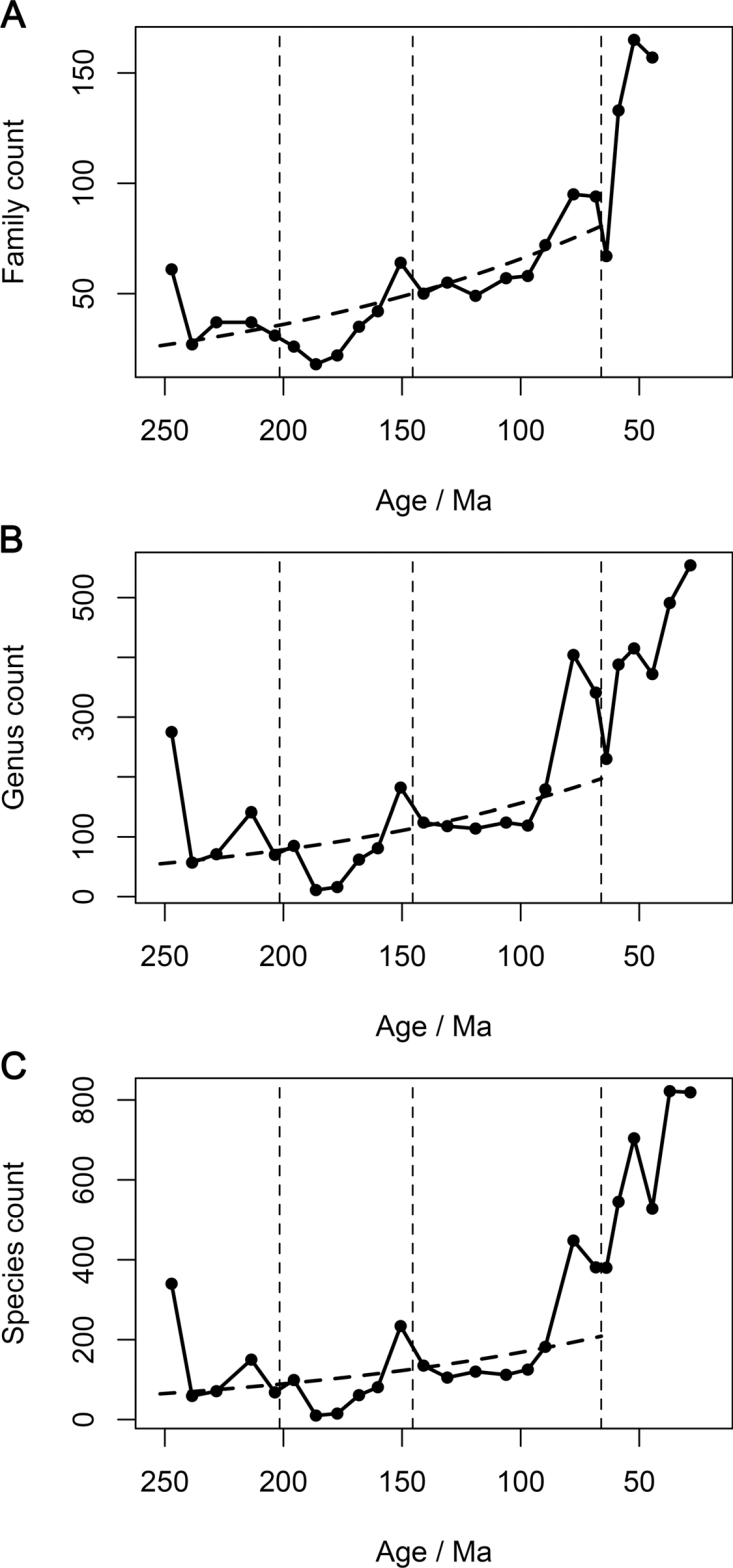
Global counts of non-marine tetrapod taxa through time. (A) Families (from [38]), (B) genera, and (C) species. Dashed lines are general linear models predicting taxon counts from geological age in mega-annum (Ma) for the entire Mesozoic, modelling taxon counts as a Poisson distribution and using a ln() link function (coefficients in Table 1). The data displayed in this figure can be accessed at http://doi.org/10.5061/dryad.9fr76 [48].

“Face-value” observed counts of genera and species occurring globally within time bins approximating 9 million years (Myr) (S1 Table; S1 Appendix) provide little support for exponential diversity increases during the Mesozoic. These counts resemble previously reported global tetrapod family counts [12,38] in several details, including the occurrence of Paleogene diversity levels that are several times greater than those of most Mesozoic intervals (Fig 1). Furthermore, within the Mesozoic, direct counts of families, genera, and species are all highest in the final two stages, the Campanian and Maastrichtian (Fig 1). Nevertheless, counts of genera and species show different long-term patterns than counts of families.

**Table 1:** Relationships between global face-value taxon counts and geological age (= Time) during the Mesozoic based on general linear models assuming a negative binomial error distribution and ln() link function.

Model	Excluded intervals	Slope	SE (slope)	p (slope)	Intercept	Theta	N	R^2^
Families ~ Time	none	-0.00558	0.00141	<0.001*	4.57	12.9	19	0.49
Families ~ Time	K7 and K8	-0.00415	0.00157	0.008*	4.33	14.2	17	0.32
Families ~ Time	Tr1	-0.00779	0.00113	<0.001*	4.78	33.2	18	0.74
Families ~ Time	Tr1, K7 and K8	-0.00652	0.00124	<0.001*	4.58	41.1	16	0.65
Genera ~ Time	none	-0.00499	0.00309	0.106	5.51	2.11	19	0.17
Genera ~ Time	K7 and K8	-0.00121	0.00332	0.716	4.84	2.42	17	0.01
Genera ~ Time	Tr1	-0.00946	0.00280	<0.001*	5.91	3.07	18	0.45
Genera ~ Time	Tr1, K7 and K8	-0.00593	0.00305	0.052	5.33	3.46	16	0.21
Species ~ Time	none	-0.00451	0.00337	0.181	5.54	1.77	19	0.12
Species ~ Time	K7 and K8	-0.00028	0.00363	0.939	4.79	2.02	17	0.00
Species ~ Time	Tr1	-0.00966	0.00304	0.002*	5.99	2.58	18	0.42
Species ~ Time	Tr1, K7 and K8	-0.00574	0.00334	0.085	5.35	2.88	16	0.17

Interval name abbreviations are given in S1 Table. SE is the standard error, theta is the dispersion parameter of the negative binomial distribution, N is the sample size (number of intervals containing data), and R^2^ is the generalised coefficient of determination [51]. Asterisks indicate statistical significance.

General linear models assuming a negative binomial error distribution and ln() link function were used to predict global family counts from geological age across the entire Mesozoic. We found a statistically significant, negative slope that is robust to the exclusion of influential data points (Table 1), indicating a long-term trend of increasing family counts through time. By contrast, statistically significant trends in genus and species counts are only supported if the first Triassic time bin (Tr1; S1 Table), an influential data point with high leverage, is excluded (Table 1). Furthermore, the significance of this increase is largely due to the occurrence of high taxon counts in the final two time bins of the Cretaceous (K7 and K8), and the slope becomes marginally non-significant when these time bins are also excluded (Table 1). Notably, Late Triassic and latest Jurassic taxon counts also exceed those of most Cretaceous time bins. These observations indicate either that substantial species and genus diversification occurred only in the latest Mesozoic or that oversampling of latest Mesozoic terrestrial faunas has inflated face-value diversity counts for the Campanian and Maastrichtian. The latter possibility is more consistent with our further analytical results, described below. The observation that counts of lower-level taxa do not show a robust trend of Mesozoic increase (Fig 1B and 1C; Table 1) refutes the proposition that species counts should reveal the hypothesised underlying exponential nature of diversification more prominently than do studies at higher taxonomic levels [30,49,50].

**Fig 2:**
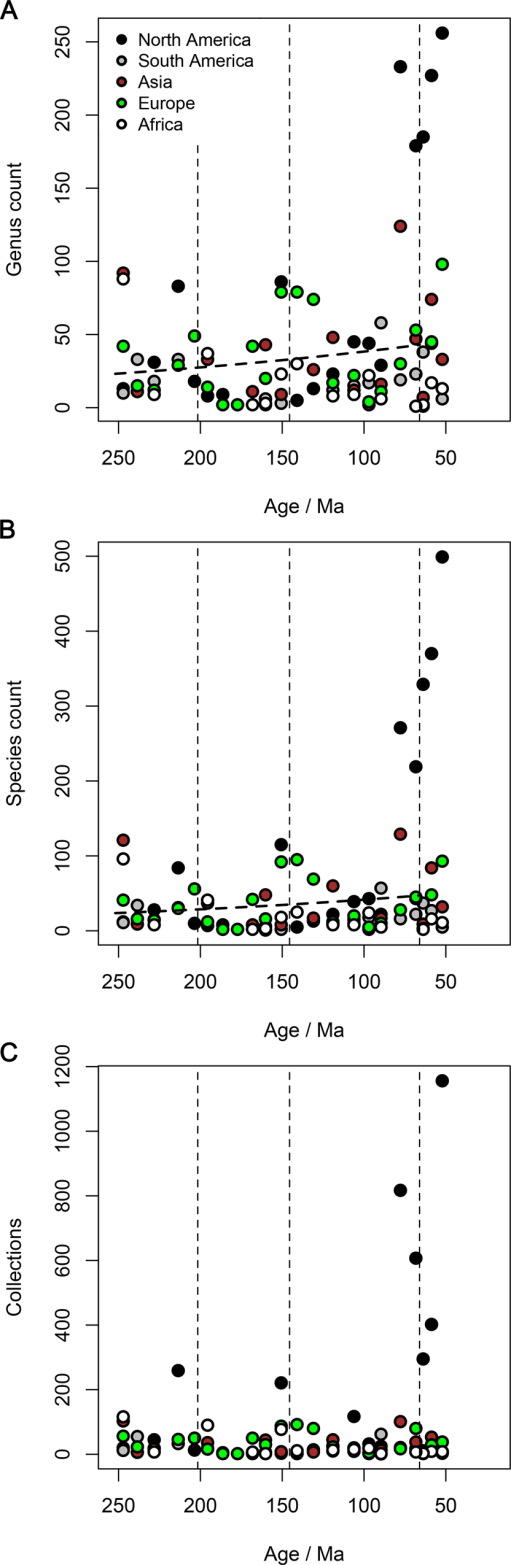
Regional counts of non-marine tetrapod taxa and fossil collections through time. (A) Genera, (B) species, (C) collections yielding non-marine tetrapod fossils. Dashed lines in A and B are general linear models predicting taxon counts from geological age for the entire Mesozoic, modelling taxon counts as a Poisson distribution and using a ln() link function (coefficients in Table 2). The data displayed in this figure can be accessed at http://doi.org/10.5061/dryad.9fr76 [48].

Pooled regional face-value genus and species counts (Fig 2) also show no evidence for a Mesozoic trend of increase. General linear models predicting these counts using geological age across the Mesozoic have non-significant slopes (Table 2). Taxon counts for the first time bin (Tr1) in Asia and Africa, and for the last two time bins (K7, K8; S1 Table) in North America are identified as influential data points with high leverage. Significant negative slopes are obtained only when the influential Tr1 data points are excluded from the analysis on their own, and not when all influential data points are excluded together (Table 2). The absence of a trend of increasing regional taxon counts through the Mesozoic is consistent with the observation that most continental regions lack any Late Cretaceous increase (Fig 2), with the exception of North America, where Campanian and Maastrichtian deposits are disproportionately well sampled, with approximately six to eight times as many collections as are known from the most intensively sampled time intervals in regions outside of North America, or two to three times as many collections as the most highly sampled other North American intervals (Fig 2C).

**Table 2:** Relationships between regional face-value taxon counts and geological age (= Time) during the Mesozoic based on general linear models assuming a negative binomial error distribution and ln() link function.

Model	Excluded intervals	Slope	SE (slope)	p (slope)	Intercept	Theta	N	R^2^
Genera ~ Time	None	-0.00259	0.00206	0.210	3.87	1.07	72	0.03
Genera ~ Time	All influentials	-0.00170	0.00201	0.396	3.52	1.32	68	0.01
Genera ~ Time	K7 and K8 influentials	0.00044	0.00196	0.823	3.27	1.25	70	0.00
Genera ~ Time	Tr1 influentials	-0.00485	0.00210	0.021*	4.12	1.13	70	0.08
Species ~ Time	None	-0.00278	0.00222	0.211	3.96	0.94	71	0.03
Species ~ Time	All influentials	-0.00185	0.00213	0.386	3.56	1.20	67	0.01
Species ~ Time	K7 and K8 influentials	0.00083	0.00210	0.694	3.24	1.12	69	0.00
Species ~ Time	Tr1 influentials	-0.00560	0.00224	0.013*	4.27	1.02	69	0.10

Interval name abbreviations are given in S1 Table. SE is the standard error, theta is the dispersion parameter of the negative binomial distribution, N is the sample size (number of intervals containing data), and R^2^ is the generalised coefficient of determination [51]. Asterisks indicate statistical significance.

#### Analytical Results: Subsampled Diversity and Paleogeographic Bias

**Fig 3:**
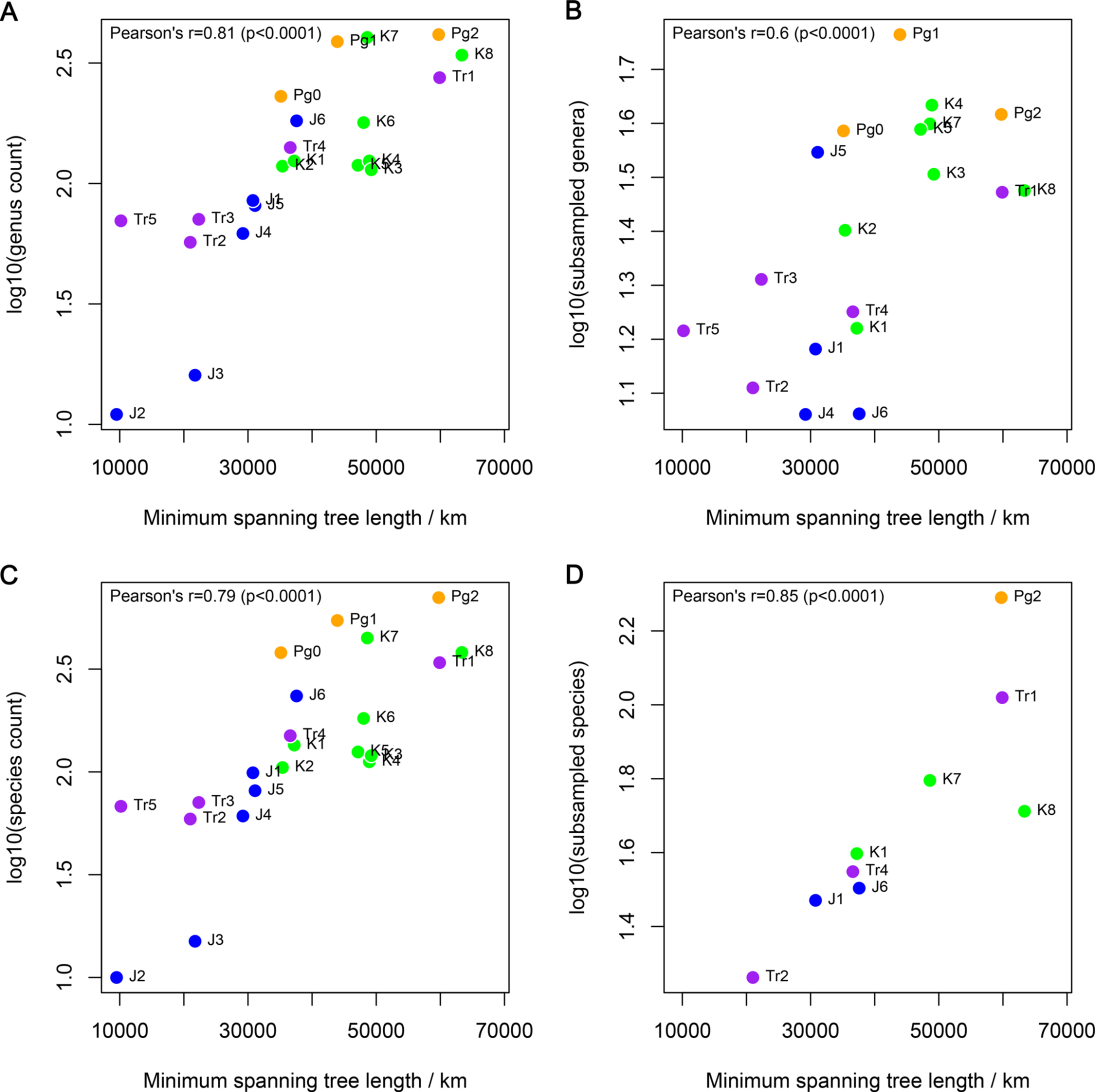
“Global” non-marine tetrapod species diversity versus paleogeographic spread of fossil localities. Using (A) genus counts, (B) subsampled genera, (C) species counts, and (D) subsampled species. Paleogeographic spreads are the minimum spanning tree lengths in km. Subsampled values were obtained using a quorum of 0.4. Correlation coefficients and *p*-values from Pearson’s correlation tests are reported in the top-left of each panel. Abbreviated interval names are given in full in S1 Table. The data displayed in this figure can be accessed at http://doi.org/10.5061/dryad.9fr76 [48].

Uneven fossil record sampling may substantially bias directly counted diversity patterns. To address this, we applied equal-coverage or shareholder quorum subsampling (SQS) to standardise sampling among time bins. Most subsampling approaches, including SQS, require that geographic spread is held approximately constant to allow meaningful comparison of regional gamma diversities (e.g., [52]). However, both directly counted and subsampled patterns of “global” richness are biased by differences in paleogeographic sample spread, estimated as the length of the minimum spanning tree uniting the paleocoordinates of non-marine tetrapod-bearing localities for each interval (Fig 3). This bias is evidenced by strong, statistically significant, positive correlations, a pattern that is similar to geographic bias in the marine invertebrate [53] and Miocene North American mammal [54] fossil records and which explains 72% of the variance in global subsampled species counts (Fig 3D) and 62% of the variance in directly observed species counts (Fig 3C).

The minimum spanning trees for global fossil localities circumscribe planetary spatial scales from 9,500 km to 68,800 km, with a range that is comparable to the circumference of the Earth (~40,075 km). At this scale, the correlation between paleogeographic sample spread and diversity could result from either or both of two possibilities: (1) A direct bias model, in which the correlation is due to variation in the number of distinct global regions for which data are available in each time bin (assuming that each region existed even during intervals in which it is unsampled). (2) A “common cause” model, in which processes such as sea level changes, orogeny, and rifting determine the absolute sizes of individual regions, thereby determining both paleogeographic sample spread and species richness via species-area effects (e.g., [55]). Both models assume that a species-area relationship exists (i.e., that available land area constrains species diversity). They differ in that the direct bias model assumes that differences in the area sampled for each interval result from bias, whereas the common cause model assumes that they result from actual changes in the ancient Earth system.

We tested between these alternatives by examining the correlations and partial correlations between taxonomic richness measures and three measures of the geographic distribution of localities: (1) counts of geographically “long” branches of our minimum spanning trees, representing the number of distinct global regions sampled; (2) counts of geographically “short” branches of our minimum spanning trees, representing the addition of fossil localities to regions that have already been sampled; and (3) the summed lengths of geographically “short” branches of our minimum spanning trees, representing the geographic spread of localities within local regions. There is no clear distinction between “short” and “long” branches in our minimum spanning trees. However, histograms of the frequency distributions of branch lengths in the intervals with the greatest total geographic spread suggest that a frequency transition occurs somewhere between 100 and 1,000 km (S1 Fig), so we performed analyses at several “threshold” branch lengths between these values, and up to 2,000 km. In general, counts of long branches are strongly and significantly correlated with both of our “short branch” measures (Table 3), indicating that intervals with more sampled regions also have greater total local sampling.

**Table 3:** Correlations and partial correlations of diversity measures and measures of local and global geographic spread.

		Pearson's r and *p*-value (in parentheses) at thresholds				
Model	N	100 km	200 km	500 km	1,000 km	2,000 km
local spread ~ regions	9	0.8 (0.009)	0.88 (0.002)	0.91 (0.001)	0.89 (0.001)	0.79 (0.011)
localities ~ regions	9	0.64 (0.066)	0.73 (0.025)	0.69 (0.039)	0.5 (0.168)	0.72 (0.028)
log10 (subsampled species) ~ regions	9	0.78 (0.013)	0.83 (0.005)	0.76 (0.017)	0.66 (0.054)	0.82 (0.007)
log10 (subsampled species) ~ local spread	9	0.43 (0.254)	0.54 (0.134)	0.69 (0.042)	0.76 (0.019)	0.68 (0.042)
log10 (subsampled species) ~ localities	9	0.75 (0.021)	0.75 (0.019)	0.76 (0.018)	0.76 (0.017)	0.76 (0.017)
log10 (subsampled species) ~ regions | local spread	9	0.82 (0.001)	0.9 (<0.001)	0.45 (0.211)	-0.04 (0.912)	0.62 (0.051)
log10 (subsampled species) ~ local spread | regions	9	-0.54 (0.112)	-0.74 (0.007)	-0.02 (0.958)	0.5 (0.163)	0.09 (0.818)
log10 (subsampled species) ~ regions | localities	9	0.6 (0.065)	0.63 (0.049)	0.5 (0.156)	0.49 (0.168)	0.6 (0.066)
log10 (subsampled species) ~ localities | regions	9	0.52 (0.137)	0.39 (0.305)	0.5 (0.161)	0.66 (0.03)	0.43 (0.237)
local spread ~ regions	19	0.78 (<0.001)	0.82 (<0.001)	0.82 (<0.001)	0.83 (<0.001)	0.77 (<0.001)
localities ~ regions	19	0.66 (0.002)	0.69 (0.001)	0.62 (0.005)	0.5 (0.028)	0.6 (0.007)
log10 (subsampled genera) ~ regions	19	0.62 (0.004)	0.66 (0.002)	0.62 (0.004)	0.49 (0.033)	0.6 (0.007)
log10 (subsampled genera) ~ local spread	19	0.31 (0.196)	0.41 (0.084)	0.51 (0.027)	0.61 (0.006)	0.53 (0.019)
log10 (subsampled genera) ~ localities	19	0.41 (0.079)	0.42 (0.073)	0.43 (0.069)	0.43 (0.065)	0.43 (0.064)
log10 (subsampled genera) ~ regions | local spread	19	0.64 (0.001)	0.62 (0.002)	0.42 (0.061)	-0.03 (0.892)	0.35 (0.136)
log10 (subsampled genera) ~ local spread | regions	19	-0.36 (0.118)	-0.3 (0.21)	-0.02 (0.943)	0.42 (0.065)	0.14 (0.583)
log10 (subsampled genera) ~ regions | localities	19	0.51 (0.018)	0.56 (0.007)	0.51 (0.019)	0.35 (0.135)	0.47 (0.032)
log10 (subsampled genera) ~ localities | regions	19	0 (0.994)	-0.05 (0.829)	0.07 (0.793)	0.25 (0.31)	0.12 (0.637)
local spread ~ regions	22	0.81 (<0.001)	0.84 (<0.001)	0.85 (<0.001)	0.84 (<0.001)	0.82 (<0.001)
localities ~ regions	22	0.67 (0.001)	0.68 (<0.001)	0.63 (0.002)	0.5 (0.017)	0.58 (0.005)
log10 (counted species) ~ regions	22	0.8 (<0.001)	0.82 (<0.001)	0.75 (<0.001)	0.73 (<0.001)	0.79 (<0.001)
log10 (counted species) ~ local spread	22	0.76 (<0.001)	0.77 (<0.001)	0.84 (<0.001)	0.82 (<0.001)	0.78 (<0.001)
log10 (counted species) ~ localities	22	0.75 (<0.001)	0.75 (<0.001)	0.76 (<0.001)	0.76 (<0.001)	0.76 (<0.001)
log10 (counted species) ~ regions | local spread	22	0.48 (0.018)	0.49 (0.013)	0.13 (0.558)	0.14 (0.543)	0.43 (0.039)
log10 (counted species) ~ local spread | regions	22	0.32 (0.134)	0.27 (0.226)	0.56 (0.003)	0.56 (0.003)	0.38 (0.073)
log10 (counted species) ~ regions | localities	22	0.61 (0.001)	0.63 (<0.001)	0.54 (0.005)	0.62 (0.001)	0.67 (<0.001)
log10 (counted species) ~ localities | regions	22	0.47 (0.019)	0.46 (0.024)	0.56 (0.003)	0.67 (<0.001)	0.61 (0.001)
local spread ~ regions	22	0.81 (<0.001)	0.84 (<0.001)	0.85 (<0.001)	0.84 (<0.001)	0.82 (<0.001)
localities ~ regions	22	0.67 (0.001)	0.68 (<0.001)	0.63 (0.002)	0.5 (0.017)	0.58 (0.005)
log10 (counted genera) ~ regions	22	0.82 (<0.001)	0.84 (<0.001)	0.78 (<0.001)	0.76 (<0.001)	0.81 (<0.001)
log10 (counted genera) ~ local spread	22	0.77 (<0.001)	0.79 (<0.001)	0.85 (<0.001)	0.84 (<0.001)	0.81 (<0.001)
log10 (counted genera) ~ localities	22	0.72 (<0.001)	0.73 (<0.001)	0.74 (<0.001)	0.74 (<0.001)	0.74 (<0.001)
log10 (counted genera) ~ regions | local spread	22	0.52 (0.009)	0.52 (0.008)	0.2 (0.38)	0.2 (0.382)	0.44 (0.032)
log10 (counted genera) ~ local spread | regions	22	0.33 (0.131)	0.29 (0.19)	0.56 (0.003)	0.56 (0.003)	0.43 (0.036)
log10 (counted genera) ~ regions | localities	22	0.65 (<0.001)	0.68 (<0.001)	0.6 (0.001)	0.67 (<0.001)	0.7 (<0.001)
log10 (counted genera) ~ localities | regions	22	0.41 (0.049)	0.4 (0.06)	0.5 (0.011)	0.64 (<0.001)	0.57 (0.003)

All three of our geographic distribution measures are strongly and significantly correlated with face-value counts of both genera and species over the full range of thresholds examined (Table 3). Counts of long branches retain strong and significant relationships with taxon counts when conditioned on counts of short branches, but they have a non-significant relationship with taxon counts when conditioned on the summed lengths of short branches at thresholds of 500 and 1,000 km. Counts of long branches have strong and significant or near significant (*p* = 0.054; subsampled species | threshold = 1,000 km) correlations with subsampled species and genus diversity estimates (Table 3), and retain substantial power to explain subsampled diversity estimates (R^2^ > 0.45 [species]; R^2^ > 0.35 [genera]) when conditioned on both of our of short branch measures. The variance explained by partial correlation for long branch counts is greater than that for short branch measures when conditioned on counts of long branches in almost all cases, with exceptions only at a threshold of 1,000 km (Table 3).

The results described above suggest that both directly counted and subsampled diversity measures are at least partly explained by the number of distinct global regions sampled. This indicates that sampling bias determines global richness patterns in the tetrapod fossil record. In fact, these “global” patterns result from a heterogeneous assemblage of regional patterns, and the presence of sample spread bias seriously undermines the use of global fossil occurrence data as an adequate summary of standing diversity through time. Therefore, we report subsampling results for contiguous continental regions defined in S2 Table (S1 Appendix), and not for the entire planet taken as a whole.

**Fig 4:**
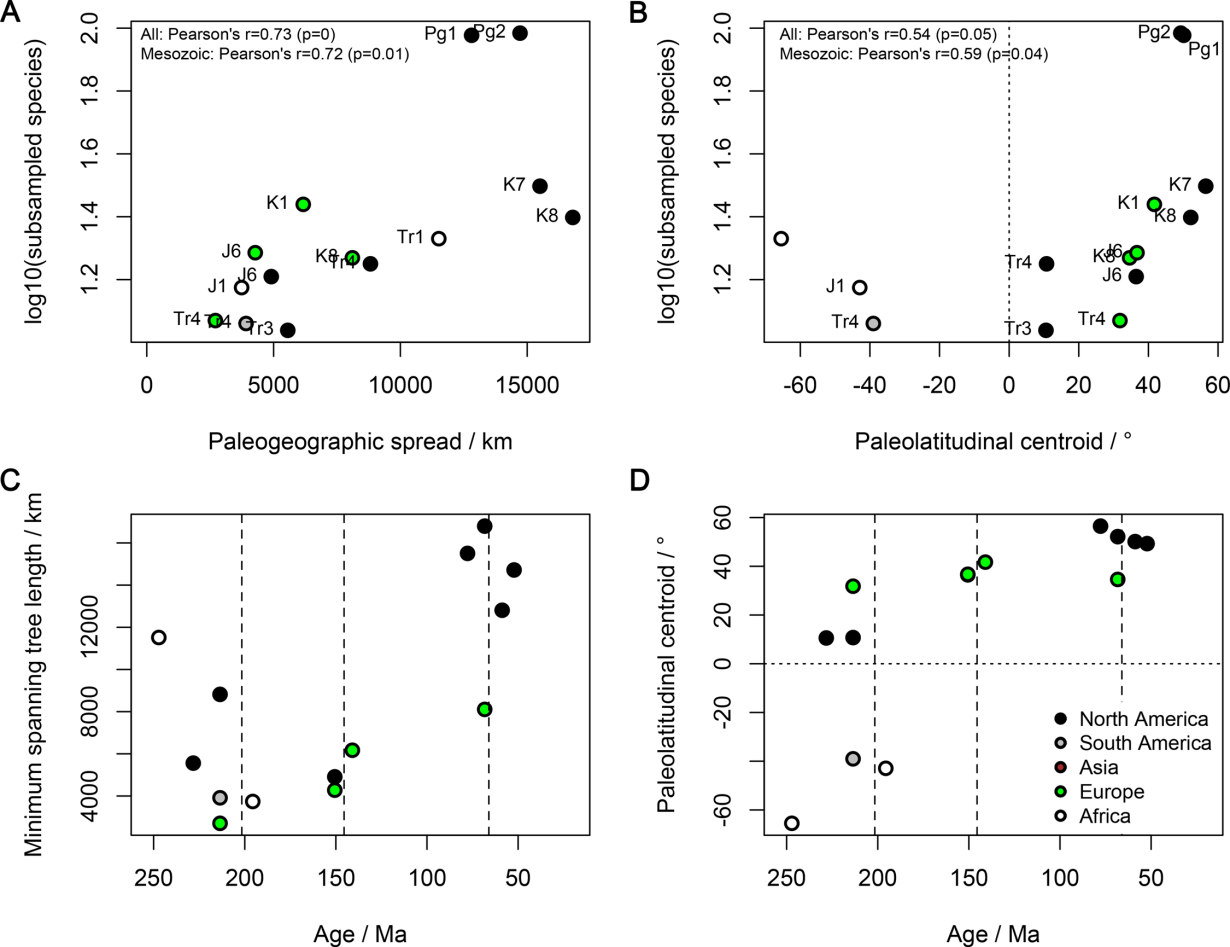
Regional subsampled non-marine tetrapod species diversity versus paleogeographic spreads and paeolatitudinal centroids of fossil localities. (A) Regional subsampled species diversity versus paleogeographic spread. (B) Regional subsampled species diversity versus paleolatitudinal centroid (positivised value). (C) Regional paleogeographic spreads versus geological age (Ma). (D) Regional paleolatitudinal centroids versus geological age (Ma). Paleogeographic spreads are the minimum spanning tree lengths in km. Subsampled values were obtained using a quorum of 0.4. Correlation coefficients and *p*-values from Pearson’s correlation tests are reported in the top-left panels A and B. Abbreviated interval names are given in full in S1 Table. The data displayed in this figure can be accessed at http://doi.org/10.5061/dryad.9fr76 [48].

No individual region is represented sufficiently well to provide a continuous time series of subsampled diversity. However, variation in paleogeographic sample spread and paleolatitude among regions and through time could bias a pooled regional analysis. To investigate this, we compared regional subsampled diversities to regional minimum spanning tree lengths and paleolatitudinal centroids (median paleolatitudes of collections) across the Mesozoic, using general linear models with a Gaussian error distribution and ln() link function (Fig 4; Table 1). In univariate comparisons, subsampled diversity has significant positive relationships with geographic spread, but not with absolute paleolatitude, and a significant negative relationship with geological time (Table 4), indicating higher subsampled diversity estimates in younger time bins. Comparisons of the AICc-weights [56] of regression models including combinations of time, geographic spread and paleolatitude as explanatory variables indicate that a univariate model explaining regional subsampled diversities using only geographic spread (AICc-weight = 0.50) is more likely than one using geological time (= 0.12), and substantially more likely than one using paleolatitude (= 0.02). The second best model (AICc-weight = 0.18), is one in which time is included as an explanatory variable together with geographic spread in a multiple regression. In this model, the slope of geological time is reduced to approximately half of its value in a univariate model (Table 4: from -0.00335 to -0.00148) and is non-significant, whereas the slope of geographic spread is reduced by less, and becomes marginally non-significant (Table 4: from 0.0000489 to 0.0000356). The slope of paleolatitude is non-significant in all models (Table 4).

**Table 4:** Relationships of geographic spread (minimum spanning tree length in km), time and palaeolatitude (positivised median regional paleolatitude) with subsampled regional species diversities (quorum = 0.4) based on general linear models assuming a Gaussian error distribution and ln() link function.

Dependent variable	Explanatory variable(s)	Interval	AICc	AICc weight	Slope	SE (slope)	p (slope)	Intercept	N	R^2^
Subsampled diversity	Geographic spread	Mesozoic	75.9	0.504	0.0000489	0.0000135	0.00476*	2.54	12	0.58
Subsampled diversity	Time	Mesozoic	78.8	0.118	-0.00335	0.00123	0.0215*	3.46	12	0.45
Subsampled diversity	Palaeolatitude	Mesozoic	82.1	0.023	0.00512	0.0029	0.108	2.84	12	0.25
Subsampled diversity	Geographic spread	Mesozoic	78	0.176	0.0000356	0.0000172	0.0676	2.88	12	0.63
	Time				-0.00148	0.00132	0.29			
Subsampled diversity	Geographic spread	Mesozoic	78.2	0.16	0.0000421	0.0000145	0.0175*	2.56	12	0.63
	Palaeolatitude				0.00212	0.00203	0.323			
Subsampled diversity	Time	Mesozoic	82.4	0.02	-0.00309	0.00194	0.146	3.41	12	0.45
	Palaeolatitude				0.000743	0.00362	0.842			

SE is the standard error, N is the sample size (number of intervals containing data), and R^2^ is the generalised coefficient of determination [51]. Asterisks indicate statistical significance.

The correlation of subsampled diversity with within-region geographic sample spread could be explained in one of two possible ways, as discussed above for “global” diversity. The first is that variation in paleogeographic spread among regions and intervals systematically biases our subsampled diversity estimates, artifactually enhancing the trend towards increasing subsampled diversity through time, and increasing the scatter of subsampled diversities. The second is that the correlation between paleogeographic spread and subsampled regional diversity results from a common-cause process, in which a third variable such as continental flooding drives both genuine biodiversity and changes in regional sample spreads, as is well-documented in shallow marine biotas (e.g., [55]). We cannot presently distinguish between these two hypotheses, so we discuss results for the relationship between time and subsampled diversity estimates under both models.

### Analytical Results: Subsampled Diversity Patterns

**Fig 5:**
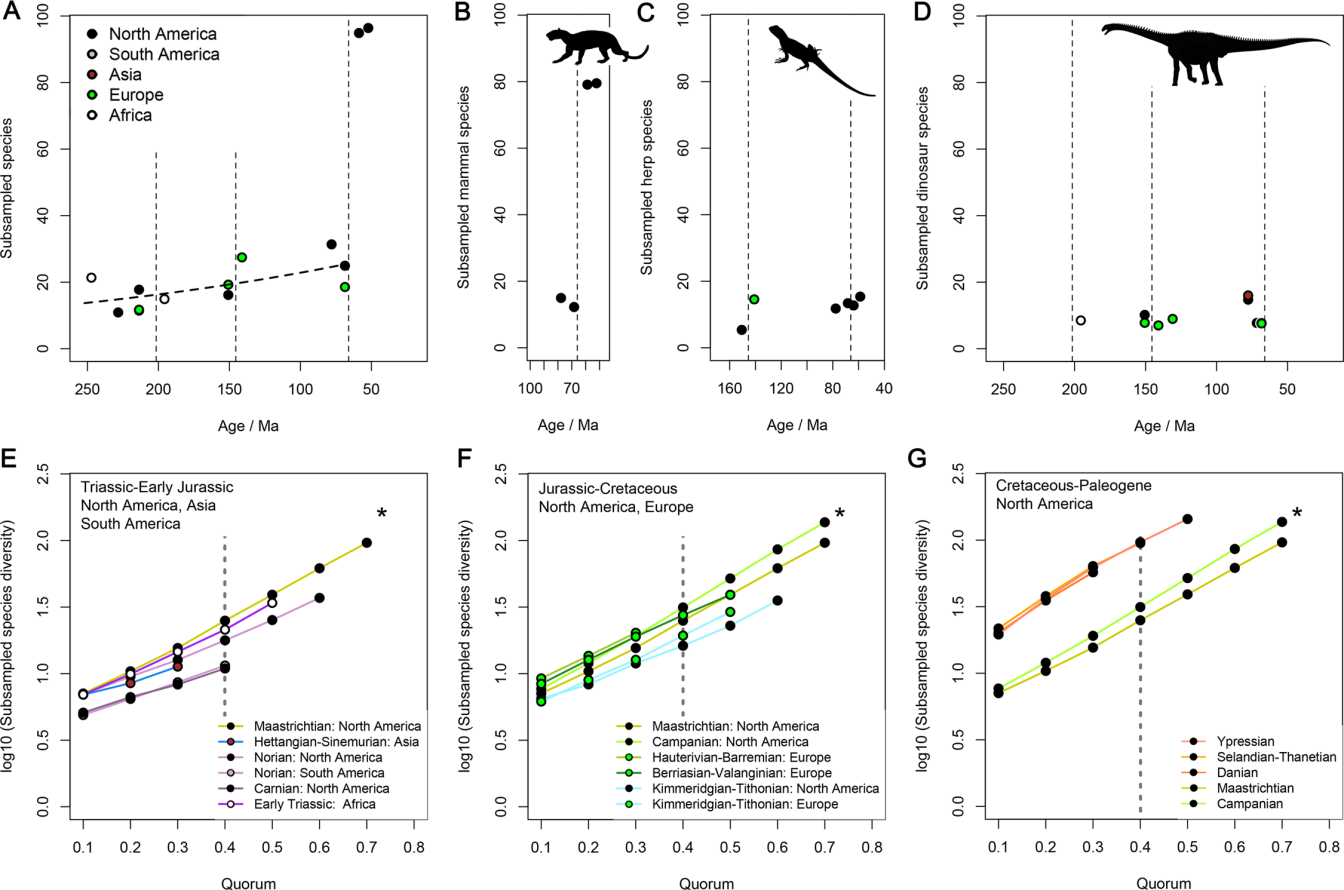
Subsampled non-marine tetrapod species diversity. (A) Subsampled species diversity within continental regions for a quorum of 0.4. The dashed line in A is the general linear model predicting subsampled regional diversity from geological age for the entire Mesozoic, modelling taxon counts as a Gaussian distribution and using a ln() link function (coefficients in Table 4). (B–D) Subsampled diversities for mammals (B), herps (C; non-mammalian, non-dinosaurian tetrapods), and dinosaurs (D). (E–G) Subsampling curves for (E) the Triassic–Early Jurassic of North America, Asia, and South Africa, (F) the Jurassic–Cretaceous of North America and Europe, and (G) the Cretaceous–Palaeogene of North America. The vertical, dashed grey lines in E–G indicate the target quorum of 0.4. An asterisk is placed in the same location of plots E–G to aid comparison. The data displayed in this figure can be accessed at http://doi.org/10.5061/dryad.9fr76 [48].

Regional subsampling results indicate a protracted interval of only limited increases in standing diversity spanning the entire Mesozoic (Fig 5A). Similar subsampled diversity estimates were obtained for widely separated time intervals such as the Maastrichtian (72.1–66 mega-annum [Ma]) and Kimmeridgian–Tithonian (157–145 Ma) of Europe, and the Kimmeridgian–Tithonian and Norian (228–208 Ma) of North America (Fig 5A and 5E–5G). The sporadic availability of data that is rich enough for rigorous diversity estimates makes it difficult to infer short-term patterns of change in standing diversity, although Cretaceous values seem generally higher than those of the Triassic and Jurassic. Nevertheless, we are able to estimate the resultant long-term net diversification rate using general linear models (Table 4), acknowledging that this represents a simplification of potentially more complex short-term patterns.

The general linear model using geological time to explain subsampled diversity, pooled across geographic regions for the entire Mesozoic, demonstrates only a very weak, but significant slope (Fig 5A; Table 4; *p* = 0.02), indicating a trivial net diversification rate of 0.00335 ln(species)/Myr (±2 standard errors yields 0.00089–0.00581 ln(species)/Myr). This implies an expected increase in species richness of 0.637 ln(species), or 89% over *c*.190 Myr (±2 standard errors yields 18%–202%; and when within-region geographic spread is considered to be a bias the net diversification rate is reduced to 0.00148 ln(species)/Myr, predicting a diversity increase of 32%; Table 4). This expected value is equivalent to less than one net speciation per lineage, and comparable to three standard deviations of the regression residuals (s.d. = 0.28). Therefore, short-term diversity fluctuations and statistical counting error have a similar magnitude to our estimate of the long-term expansion of diversity through the Mesozoic. The failure of short-term and inter-regional diversity variations to sum to a greater long-term change would be direct evidence of diversity dependence if we could demonstrate that the proportion of these short-term variations attributable to counting error was low [22,57].

Our estimated long-term diversification rate of 0.00335 ln(species)/Myr is particularly striking in context of the increase in tetrapod diversity that must have occurred during the *c*.130 million years prior to our study interval, from the Late Devonian origin of tetrapods to the early Mesozoic, which entailed substantially more than a doubling of diversity (e.g., [38,58,59]). This can be demonstrated by approximation, assuming that Late Permian diversity was comparable to Early Triassic diversity, which is estimated from the general linear model of subsampled diversity on time as 2.62 ln(subsampled species). The transition from ln(1) to ln(2.62) over 130 million years implies a Paleozoic long-term net diversification rate of 0.0202 ln(species)/Myr, which would generate more than a 40-fold increase in diversity over 190 Myr of Mesozoic time. This estimate is conservative: it could only increase if Late Permian diversity was higher than that of the Early Triassic, as is possible due to the occurrence of the Permian/Triassic boundary mass extinction event (e.g., [60]). The overall pattern therefore seems to be one of substantial reductions in the long-term net diversification rate of tetrapods during the Paleozoic and Mesozoic, representing the first 87% of their evolutionary history.

Furthermore, equation A25 of reference [61] (see Materials and Methods: Raup Equation) gives the expected diversity of a clade after a specified time under specified birth and death rates, conditioned on the observation that the clade survived until that time had elapsed. We assumed that the tetrapod crown group originated 100 Myr earlier in the Late Carboniferous, and then specified a net diversification rate of 0.00335 ln(species)/Myr (conservatively assuming the higher diversification rate implied by a direct bias model), and per-lineage death rates of 0.10, 0.15, 0.20, 0.25, and 0.30 ln(extinctions)/Myr (centred on values estimated for Cenozoic North American mammals [22]). This gives expected Early Triassic regional diversities of 13.3, 19.2, 25.2, 31.1, and 37.0 species. We do not know the actual (rather than observed or subsampled) regional diversities of any studied intervals. However, these expected values of Late Palaeozoic regional diversity obtained under the estimated Mesozoic net diversification rate are lower than the face-value regional species counts of Tr1 in Asia (121 species), Africa (96 species), and Europe (41 species), and of Tr2 in South America (34 species). The diversity counts for these relatively well-sampled regions are not corrected for the possible existence of multiple chronofaunas that could cause counts to exceed the standing diversity at any single time horizon, and they immediately follow the Permian/Triassic extinction event rather than representing Late Permian diversity.

An abrupt and substantial increase in regional subsampled diversity is apparent in the earliest Paleogene, following the end-Cretaceous mass extinction 66 Mya (Fig 5A). This increment cannot be explained by bias due to paleogeographic sample spread, which does not change over the boundary (Fig 4C). It results entirely from an increase in mammalian species diversity (Fig 5B) [62,63], which is disproportionately large compared to the loss of dinosaur diversity (Fig 5D). The diversity of non-dinosaurian, non-mammalian tetrapods (“herps”; Fig 5C) does not change substantially over the Cretaceous/Paleogene (K/Pg) boundary on the temporal resolution of our study, although a major, short-term K/Pg turnover certainly occurred among herps, including squamates [64]. Nevertheless, our subsampling results tentatively suggest a doubling of herp diversity around the Jurassic/Cretaceous boundary (Fig 5; S1 Fig; S1 Appendix), consistent with patterns of subsampled fossil turtle diversity [65].

One possibility is that a taxonomic restructuring of terrestrial ecosystems at the K/Pg boundary rapidly established a new dinosaurian/mammalian diversity equilibrium that substantially exceeded the Mesozoic baseline. Such rapid equilibration could only be possible under strong diversity-dependence of diversification rates (e.g., [28,46]). However, mammals, which have increased proportional representation in Cenozoic ecosystems, possess complex teeth, allowing more precise taxonomic identifications from highly fragmentary material than can be diagnosed in fossils of the other highly diverse extant clades (lissamphibians, squamates, and birds), and potentially also Mesozoic dinosaurs. The relatively greater ability to diagnose mammalian species based on fragmentary fossil remains compared to non-mammalian tetrapods is evident in our results: the ratio of mammalian species to species of lissamphibians plus squamates on Earth today is about 1:3, but our subsampled diversity estimates from fossil data yield a ratio of >5:1 in the Paleocene. This suggests that an increase in the proportion of mammalian species within the total terrestrial tetrapod fauna should result in an increase in apparent species diversity in the fossil record, even in the absence of any change in actual tetrapod diversity. It is therefore possible that this apparent Paleocene increase in diversity at least partly reflects a change in the nature of terrestrial vertebrate taxonomy, and is not necessarily a genuine evolutionary phenomenon.

### Alpha Diversity Patterns

**Fig 6:**
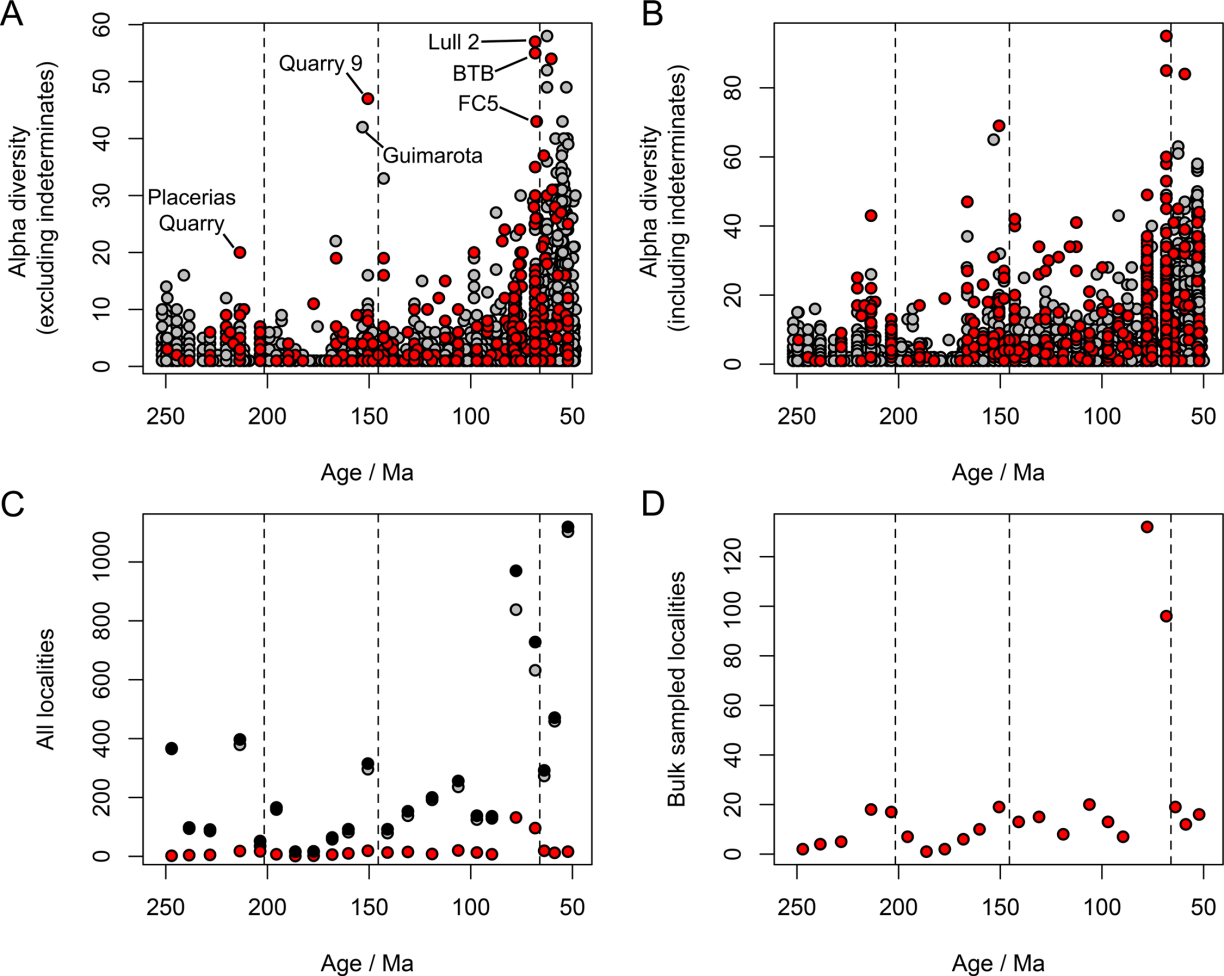
Within-locality alpha diversities of non-marine tetrapods and sampling methods against geological age. (A) Alpha diversity excluding records that are indeterminate at the species level. (B) Alpha diversity including records that are indeterminate at the species level. (C) Per-interval global locality counts (black). (D) Per-interval global bulk sampled locality counts. In all panels, localities that have not been bulk sampled for microvertebrate remains are shown in grey and localities that have been bulk sampled are shown in red. The data displayed in this figure can be accessed at http://doi.org/10.5061/dryad.9fr76 [48].

Patterns of tetrapod alpha diversity, measured as counts of taxa found within individual fossil localities, are consistent with slow Mesozoic diversification among non-flying tetrapods. Specimens that are taxonomically determinate at the species level are present in 4,357 Mesozoic localities. Of these, just a handful of localities yield substantially greater species counts than most others, including the Late Triassic Placerias Quarry of North America (e.g., [66]), Late Jurassic Como Bluff Quarry 9 of North America (e.g., [67]) and Guimarota Mine of Portugal [68], and the Late Cretaceous Lull 2 Quarry and Bushy Tailed Blowout [69] of North America (Fig 6A). The rare and sporadic occurrence of maximally-diverse fossil localities presents a challenge concerning our ability to resolve patterns of local diversity in the fossil record. Nevertheless, the diversities of these maximally diverse localities increases approximately 3-fold through the Mesozoic, or 2-fold if specifically indeterminate occurrences, which can represent the occurrences of distinct clades and are therefore relevant to diversity counts, are included (Fig 6B). These values are comparable to the diversity increase estimated from regional subsampled diversities.

It is notable that the maximal within-locality counts generally occur within those intervals containing the greatest numbers of localities such as the Norian (Triassic 4), Kimmeridgian–Tithonian (Jurassic 6), Campanian and Maastrichtian (Cretaceous 7 and 8), suggesting that the intensity of fossil collection activities plays a role in determining the apparent diversity of local communities sampled in the fossil record. Notably, almost all the localities exhibiting high species richness have been intensively bulk sampled for microvertebrate remains—the highest maximal species richness occurs in the latest Cretaceous (Campanian and Maastrichtian) North American localities, which have been intensively bulk sampled (Fig 6D). At present, within-locality taxon counts do not suggest any increase in diversity during the early Cenozoic.

The results of our alpha diversity analyses should be treated as “first pass” estimates that should be investigated in more detail by future studies, because (1) we did not apply subsampling methods, (2) we did not consider potential environmental or paleogeographical differences between these localities that might affect diversity counts, (3) we did not study the specimens known from these localities to determine the minimum taxon count based on unreported or undiagnosed material, and (4) we did not quantify biases resulting from the likely increased ability of taxonomists to identify fragmentary specimens belonging to extant clades—a bias that could cause relative underestimation of alpha diversity in the Triassic, preceding the origins of most tetrapod crown groups.

### Near-Stasis in Mesozoic Tetrapod Diversification

The Mesozoic–early Cenozoic has previously been regarded as an episode of unbounded diversification, culminating in substantially increased tetrapod diversity on land [2,10–13]. In contrast to this paradigm, our analyses indicate less than a doubling of tetrapod diversity through the Mesozoic, and imply a near-zero long-term net diversification rate. Substantial increases in regional tetrapod diversity were absent from the entire Mesozoic, both for directly counted and subsampled fossil species (Figs 1C and 5). Furthermore, a possible dramatic expansion of tetrapod diversity occurred in the early Paleogene. Our conclusions are strongest if differences in paleogeographic sample spread among regions and intervals are considered to be a bias, in which case Mesozoic regional tetrapod diversity is estimated as being almost static on long time scales (Table 4). Furthermore, first-pass maximal alpha diversity estimates also indicate slow diversification rates (Fig 6), demonstrating that similar patterns occur at local and regional geographic scales. This is consistent with, though not conclusive for, the hypothesis that ecological constraints within local communities could slow diversity increases and thereby regulate diversity at larger scales [28,70]. Our results do not exclude the possibility that an increase in the number of distinct biogeographic regions due to continental fragmentation during the Cretaceous resulted in a greater increase in global diversity than that seen in regional diversities.

Our estimated long-term net diversification rates of 0.00335 or 0.00148 ln(species)/Myr are 1–2 orders of magnitude less than those reported from studies of extant tetrapods (e.g., [1,6]). This difference is unlikely to be explained by underestimation of absolute biodiversity resulting from the incompleteness of the fossil record: estimated diversification rates rely only on accurate inference of relative, not absolute, changes in diversity through time; although we cannot altogether rule out any contribution of fossil record biases (e.g., the possibility that preservational biases could mask an increase in the diversity of small-bodied taxa). Regardless of fossil record biases, a difference between net diversification rates estimated from fossils and those from extant taxa might be expected, because even the richest phylogenies of living taxa lack information on the contributions of entirely extinct clades to diversity dynamics [23,71,72]. Specifically, the contributions of extinct clades and stem groups to total extinction rates cannot easily be estimated from extant-only datasets, and the proportion of entirely extinct clades is likely to increase systematically further back in time from the present. This should cause over-estimation of net diversification rates within inclusive and ancient clades such as Tetrapoda based on the study of living taxa alone. The discrepancy between Mesozoic diversification rates inferred from fossils and diversification rates inferred from living tetrapod phylogenies could also be explained if Cenozoic diversification rates (which are the primary object of inference from living tetrapod phylogenies) substantially exceeded those of the Mesozoic.

Another explanation is plausible if tetrapod subclades show waxing/waning dynamics, as documented in invertebrate genera and mammalian families [23,73,74]. If the dynamics of subclades were asynchronous, whether this were due to diversity dependent interactions [25,75,76], variable environmental tolerances [77], or stochasticity, then the large diversity increases resulting from the waxing phases leading to speciose modern groups could be balanced on long timescales by the waning dynamics of groups that are extinct or depauperate today. This must have occurred in Cenozoic mammals, which show static and diversity-dependent diversification on large scales [22], which apparently results from a zero-sum game among smaller clades that individually exhibit waxing/waning dynamics [23,25].

Near-stasis in Mesozoic tetrapod diversification could be explained by any of three prominent hypotheses: (1) diversity-dependence of diversification rates, or “equilibrial” models, under which speciation and extinction rates become balanced at equilibrial diversity [15,26,27]; (2) the possibility of relatively stable long-term environments during the Mesozoic, which could lead to nearly static diversity under Vrba’s Turnover Pulse hypothesis [77]; or (3) a “damped exponential” model, in which unconstrained diversification is held in check by frequent, stochastic downwards perturbations [2,37,50]. Determining which of these alternatives, if any, provides a good explanation of the pattern requires further work, and we discuss each of them below.

#### Diversity dependence

The concept of diversity-dependence has been influential in the development of paleontological studies of diversification [15–17] and received significant recent attention from evolutionary biologists studying extant groups [21,28,32]. Recently, diversity dependence has been statistically demonstrated among those vertebrate and non-vertebrate groups that have sufficiently rich fossil records [15–25]. We note that “equilibrial” diversity is attained when the balance of speciation and extinction rates results in an approximately zero net diversification rate, as seen in our analyses. This need not imply that ecosystems are absolutely “saturated,” with all their niches filled. Slow increases in diversity equilibria are possible under a “damped increase” or similar diversity-dependent models in which ecological constraints impose limits to diversity that are increased by the evolutionary discovery of new niche spaces [70].

In the absence of a complete interval-to-interval data series allowing reliable estimation of short-term diversity changes, we cannot directly demonstrate diversity-dependence of tetrapod diversification rates on land using correlation tests (e.g., [16,17,20]) or other methods [22,25]. Nevertheless, rapid recovery of regional diversities following the Cretaceous/Palaeogene extinction event is predicted by diversity-dependence, and observed in our results. Furthermore, strong correlations with physically limiting variables such as palaeogeographic area can be seen as evidence of diversity-dependence because they demonstrate that standing diversity equilibriates rapidly to the availability of environmental resources [28,46]. Indeed, land area, one example of a potentially limiting environmental resource, is a key variable in MacArthur and Wilson’s equilibrial model of island biogeography [26], which is the foundation of diversity-dependent models in paleobiology and evolution [15,27]. The correlations documented here (Table 4) demonstrate scaling of diversity with geographic area, whether the geographic area spanned by fossil localities results from geographic sampling bias or from actual changes in land mass area.

We also document that a substantial decrease in long-term net diversification rates occurred through the Paleozoic–Mesozoic. Under diversity-dependence, this could be explained by the low initial diversity of Paleozoic tetrapods (presumably a single species), which would result in high net diversification rates compared to those of Mesozoic tetrapods. Decelerating diversification rates can also occur under alternative models [78]. Of these, environment-driven bursts of diversification postulated under Vrba’s Turnover Pulse hypothesis [77] are one alternative that is relevant to fossil record studies (i.e., it is not an analytical artefact of analysing phylogenies containing only extant taxa), and long time scales (i.e., it does not invoke short-term limits to speciation such as delayed post-speciation range expansion due to physiological conservatism or reproductive interference [78]).

#### Environment-driven bursts of speciation

The Turnover Pulse Hypothesis, originally formulated to describe diversification among Neogene mammals in Africa during glacial/interglacial cycles [77], proposes that global climatic forcing influences patterns of diversification. Specifically, the appearance and removal of environmental barriers to species distributions via climate change is a prerequisite of turnover: lineages generally exhibit phenotypic stasis in the absence of turnover (i.e., a punctuated equilibrial mode occurs), and environmental oscillations past a threshold amplitude are therefore necessary to drive evolutionary innovation and diversification [77]. This model could explain the variation in long-term net diversification rates documented here in the absence of diversity-dependent interactions among clades, if Mesozoic climates and environments were relatively stable compared to those of the Paleozoic and K/Pg boundary.

Although Mesozoic climates are often inferred to have been relatively stable, the Mesozoic was not free from climatic variation, and witnessed apparently extreme climatic events such as the early Turonian thermal maximum around 93 Mya, as well as subsequent global cooling towards the end of the Cretaceous (~75–66 Mya) [79]. A key question, however, is how the timescale and amplitude of Mesozoic climate oscillations [80,81] compares to those occurring during the glacial/interglacial cycles of icehouse regimes, such as that of the late Palaeozoic (mid Carboniferous–early Late Permian; e.g., [82]), and during abrupt environmental deterioration at the end of the Cretaceous (e.g., [83]). The association between climatic variation and net diversification requires thorough investigation to address the question of whether Mesozoic climatic stability is a plausible explanation of slow net diversification rates.

#### “Damped exponential” model

The damped exponential model was quantitatively formulated to describe clades whose diversification rates depended not only on within-clade standing diversity but also on the diversities of ecologically similar clades [75,76]. This represents a form of diversity dependence similar to that modelled recently in caniform mammals by reference [25]. Nevertheless, the most frequent and recent model referred to as “damped exponential” is one in which fundamentally expansionist diversification, lacking diversity-dependence, is held in check by stochastic downward perturbations caused by frequent mass extinctions [2,37,50]. The predictions of this model, and its fit to real data, are not well constrained because it has not been subjected to significant quantitative examination. Nevertheless, it could result in small or negative long-term diversification rates across large clades, even if some individual subclades show high positive diversification rates on shorter timescales. The near-zero long-term net diversification rates recovered here during the Mesozoic indicate highly balanced speciation and extinction rates. Unlike the diversity-dependent and environment-driven models, the “damped exponential” model does not invoke any terms that specifically act to regulate diversification rates around zero. Therefore, under the “damped exponential” model, near-zero net diversification rates would be coincidental rather than expected, unless the model was modified in such a way that the timings or magnitudes of downward perturbations were diversity dependent.

#### Punctuated diversification on land

The pattern of long-term stability and post-extinction radiation reported here in fossil tetrapods, if it is not an artefact of taxonomic practice, is similar to those observed in subsampled diversity curves for marine invertebrates [18], richness counts for vascular plant species in regional paleofloras [84,85], and possibly also counts of global insect families [86]. These patterns, which are consistent with multiphase equilibrial models [27,29] and with the Turnover Pulse Hypothesis [77], suggest that substantial short-term increases in organismal diversity are infrequent and episodic at large taxonomic scales, despite their frequent occurrence at smaller taxonomic scales. There is nothing inconsistent or untoward about changes in equilibrial diversity levels through time under diversity dependence or “equilibrial models” (contra [37]; e.g., [70]). In fact, they are a central prediction of the ecological limits hypothesis, and can be explained by either the occurrence of environmental changes or the evolution of key innovations that influence ecosystem resource capacities (reviewed by [28]). For example, the Cretaceous origins of angiosperms with leaf venation features that increased photosynthetic capacities relative to gymnosperms coincides with a substantial increase in the alpha diversities of regional paleofloras [84,85,87,88]. Indeed, the mid-Cretaceous diversification of angiosperms has also been proposed to have enabled the radiations of the most diverse extant tetrapod clades (e.g., [2,3,5]).

If key innovations of the kind that substantially elevate standing diversity were sufficiently frequent, then this might allow a diversification pattern that is qualitatively “expansionist,” in which case, the rate of origination of key innovations would limit the expansion of biodiversity [29]. This proposition has been framed by an expectation that key innovations evolve frequently and that they substantially increase global species diversity [50]. Our results provide no evidence of this, suggesting instead that key innovations only occurred infrequently among Mesozoic tetrapods, or that most key innovations resulted only in minor relative increases in diversity that result only in a low long-term net rate of diversification. It is also possible that “slow fuse” lag times exist between the origins of major evolutionary innovations and the subsequent triggers (e.g., subsequent innovations or environmental triggers such as mass extinction events) that lead to exceptional diversification [89].

Flying tetrapods are not known prior to the Late Triassic but constitute a substantial portion of extant tetrapod diversity (~10,000 bird species and ~1,000 bat species). As such, it is intuitive to suggest that powered flight and invasion of the air is an exceptional key innovation. However, the lack of adequate information on patterns of bird, bat, or pterosaur richness makes it difficult to address key questions such as the timing and magnitude of increases in the diversity of flying taxa, especially among pterosaurs and flying stem-group birds, which could have been highly speciose by the Cretaceous [90,91]. Much of the diversity of extant flying groups results from Cenozoic rather than Mesozoic diversification; bats made their first fossil appearance only in the final time bin of our study interval, and phylogenomic studies suggest that most of the diversification of crown-group birds occurred during the Cenozoic [9].

Although the fossil record remains relatively silent on the standing diversities of birds and, also, amphibians [3], our study does include adequate data on other speciose modern groups: mammals and squamates. Despite the origins of diverse modern higher taxa, the standing diversity of non-flying Mesozoic tetrapods was relatively static, exhibiting a near-zero net long-term diversification rate that is not commensurate with the high living species diversities of these groups. Long-term patterns of tetrapod diversity seem to have been episodic, with long, stable intervals punctuated by major increases occurring under extreme environmental perturbations and the possible influence of exceptional, but highly rare, key innovations. This pattern contrasts with the apparently higher frequency of rapid radiations at lower taxonomic levels, and is not consistent with the expansionist paradigm of unbounded and essentially exponential diversification [2,10–14].

Macroevolutionary processes unfolding on geological timescales of hundreds of millions of years are responsible for the enormous biodiversity of living species. Diversification rates inferred on these timescales are slow compared to those inferred from molecular phylogenies of hyper-speciose living groups, and there is clearly a need to test the hypothesised evolutionary processes that might explain this discrepancy. A stronger understanding of these processes requires further interrogation of living and fossil datasets.

## Materials and Methods

### Data

Mesozoic–Ypresian tetrapods were downloaded from the Paleobiology Database (http://paleobiodb.org), accessed via Fossilworks (http://fossilworks.org) on 22 January 2015. These data represent an estimated 6,520 hours of work, of which 88% was done, or authorised by, the first five authors of the present manuscript. The major contributors, in order of effort, are M. T. Carrano, J. Alroy, R. J. Butler, P. D. Mannion, R. B. J. Benson, A. M. Rees, W. Kiessling, M. E. Clapham, F. T. Fursich, M. Aberhan, and M. D. Uhen [47].

Our work included extensive checking of the completeness of the data, which we believe is essentially an accurate documentation of the literature on Mesozoic–Ypresian tetrapod taxonomy and occurrences. The data were processed by removing ootaxa, ichnotaxa, and marine taxa using a list of the names of genera, families, and higher taxa. Together, the remaining data comprise 27,260 global tetrapod occurrences of 4,898 species in 3,323 genera, spanning almost 205 million years. All data are available at DRYAD (http://doi.org/10.5061/dryad.9fr76) [48].

### Subsampling

Equal-coverage, or shareholder quorum subsampling (SQS), tracks coverage of each subsampling pool represented by the species that have been drawn, thereby subsampling more intensively when underlying richness is higher [18,46]. The substantial advantage of SQS over other subsampling methods, such as classical rarefaction, is that it is robust to the tendency of those methods to flatten out diversity curves. A total of 10,000 subsampling trials were run in each iteration.

“Coverage” is the sum of the proportional frequencies of the species sampled (i.e., if one species constitutes 23% of occurrences within an interval, then it contributes a proportional frequency of 0.23 when it is sampled during subsampling draws), and coverage of observed data is modified to estimate the coverage of the real taxon distribution for each sample pool. This is achieved by multiplying coverage of the observed data by Good’s *u*: the proportion of occurrences representing non-singleton taxa [18,46], which is a measure of sample completeness. Each interval can therefore only be subsampled to a maximum quorum level equal to Good’s *u* for that interval, meaning fewer intervals/regions can be subsampled at higher quorum levels. For example, Fig 5G shows that the Carnian of North American has a relatively low proportion of non-singleton occurrences (<0.5) and could only be subsampled to a maximum quorum of 0.4, whereas the Maastrichtian of North America has been more completely sampled, and could be subsampled to a maximum quorum of 0.7. Results based on a minimum quorum level of 0.4 are shown in Fig 5A–5D, and other empirical analyses suggest that this level is sufficient to recover relative patterns of standing diversity [46]. Indeed, similar results were obtained using different quorum levels (Fig 5E–5G) and for genera (S1 Fig).

Singleton taxa were defined based on occurrences within collections rather than publications ([92] contra [18,46]). Entire fossil collections, containing lists of species occurrences, were drawn. Previously, exclusion of either the most common taxon or the most diverse collection from each subsampling pool was proposed as a solution to Lagerstätten effects [18,46]. Instead of doing this, we excluded the three groups with Lagerstätten-dominated records: birds, bats, and pterosaurs [93–95]. The fossil records of these groups are dominated by a different taphonomic regime than those of other tetrapod groups, and do not provide sufficient information for meaningful subsampled diversity estimation. Furthermore, the well-known Early Cretaceous Jehol Biota Lagerstätten of China [96] has thus far yielded a high reported proportion of singleton occurrences, and therefore did not achieve a sufficient quorum to be included in our analyses.

Because poorly studied spatiotemporal regions could appear well sampled for stochastic reasons, returning spuriously low subsampled diversity estimates, time bins with fewer than 20 publications were excluded from our analyses. Publications, rather than occurrences, were used as a criterion to ensure that a minimum level of taxonomic scrutiny had been applied to the fossils within each spatiotemporal region. Whenever a collection corresponding to a new publication was drawn, subsequent collections were drawn from that publication only until all or three collections from that publication had been sampled [92].

### General Linear Models

We used general linear models to estimate the coefficients of relationships between richness measures (face-value taxon counts and subsampled diversity estimates; both globally and regionally) and candidate controlling variables such as time, geographic spread and regional paleolatitudinal centroids. Models were fit using the glm() function of the stats package of R version 3.1.0 [97] for Gaussian error models and the glm.nb() function of the MASS package version 7.3.33 [98]. A negative binomial distribution was used for comparisons of face-value count data, which are over-dispersed, integer-valued, and bounded at zero. Gaussian distributions were used for subsampled diversity estimates, which are continuous-valued and do not approach zero. Because diversity is generated by the process of lineage diversification, with higher absolute total rates when more lineages are present, ln() link functions were used in all analyses. The appropriateness of these distributions was confirmed by inspection of diagnostic plots using the glm.diag.plots() function of the boot package version 1.3–16 [99], and by comparing their AICc values to those of other distributions. The explanatory power of each model was estimated in comparison to an intercept-only null model using the generalised coefficient of determination [100].

### Raup Equation

Equation A25 of reference [61] is m’_t_ = (λe^(λ-μ)t^—μ) / (λ—μ), where λ is the speciation rate per lineage million years, μ is the extinction rate per lineage million years, t is the time in million years from some arbitrary starting point, and m’_t_ is the expected paraclade diversity at time t, conditioned on the fact that the paraclade survives at least until time t.

### Correlation of “Global” Diversity with Geographic Sample Spread

We calculated minimum spanning tree lengths for each of our time bins for comparison with counted and subsampled genus and species diversities, as shown in Fig 3. A custom script in R version 3.0.2 [97] implemented the following protocol: (1) A matrix of great circle distances between pairs of fossil locality paleocoordinates was constructed for each interval. (2) This was transformed to a 3xN table containing distances between pairs of localities in rows as “locality 1,” “locality 2,” and “distance.” (3) The columns of the table were ordered from shortest to longest distance. (4) The shortest distance was added to a running total, and the locality name of locality 2 was replaced with the name of locality 1 in all instances in the table. (5) Step 4 was repeated until all locality names were equal. Log_10_-transformed richness measures were compared to measures of geographic spread using correlation tests and not general linear models because our objective was to determine the significance and strengths of relationships among variables, not to determine coefficients [100].

## Supporting Information

S1 Appendix Additional methods and results. Justification of the continental regions used and subsampled genus diversity.
(DOCX)

S1 Fig Histograms of global minimum spanning tree branch lengths (in km) for the ten intervals with the longest minimum spanning trees. Interval name abbreviations are given in S1 Table. Red dashed lines indicate 100 km and 1,000 km. The data displayed in this figure can be accessed at http://doi.org/10.5061/dryad.9fr76 [90].
(TIF)

S2 Fig Subsampled genus diversity within continental regions for a quorum of 0.4. (A) Results for all tetrapods; the dashed line is the general linear model predicting subsampled regional genus diversity from geological age for the entire Mesozoic, modelling taxon counts as a Gaussian distribution and using a ln() link function (slope = -0.003; standard error of slope = 0.0015; *p* = 0.064; intercept = 3.03). (B–D) Results for mammals (B), non-mammalian, non-dinosaurian tetrapods (“herps”) (C), and dinosaurs (D). The data displayed in this figure can be accessed at http://doi.org/10.5061/dryad.9fr76 [90].
(TIF)

S1 Table Composite 9 Myr time bins used in the present study. Pg2 ends at 48.6 Ma. Occurrences were assigned to a time bin only if their stratigraphic age uncertainty was entirely contained within that bin.
(DOCX)

S2 Table Countries included in our contiguous continental regions. (DOCX)

## Abbreviations

K/Pg: Cretaceous/Paleogene
Ma: mega-annum
Myr: million years
SQS: shareholder quorum subsampling
