# Supplementary material for: Near-Stasis in the Long-Term Diversification of Mesozoic Tetrapods
Source: PLoS Biol. 2016 Jan 25;14(1):e1002359. doi: 10.1371/journal.pbio.1002359 (PMC4726655; doi:10.1371/journal.pbio.1002359)
Supplement: S1 Appendix — Justification of the continental regions used and subsampled genus diversity. (DOCX) [file pbio.1002359.s001.docx]

**Appendix S1**

**Near-stasis in the long-term diversification of Mesozoic tetrapods**

*Roger B. J. Benson, Richard J. Butler, John Alroy, Philip D. Mannion, Matthew T. Carrano, Graeme T. Lloyd*

**Justification of the continental regions used**

Continental regions were selected by targeting contiguous, well-sampled areas of approximately comparable geographic spread (Table S2). For example, although peripheral subregions such as Cuba and Greenland are technically parts of North America by cartographical conventions, they are geographically distant to, and poorly sampled compared to, the core regions of North America (United States, Canada, Mexico). Including these peripheral regions has the effect of adding a small number of occurrences of primarily singleton taxa to the sampling pool of North America. The addition of singletons causes the core region to appear less well sampled than in fact it is, and thereby inflates subsampled diversity estimates. Japan and southern Asian countries were excluded from our Asian region on similar grounds. This consideration was not an important issue for Africa or for South America, which are only sufficiently well sampled to contribute a diversity estimate to a single interval of our study (Tr1; Africa), based on occurrences in a core of southern African countries (South Africa, Tanzania, Zambia, Namibia).

**Subsampled genus diversity**

Results obtained by subsampling genera rather than species are shown in Fig. S2. These results strongly support the absence of a strong exponential trend of Mesozoic tetrapod diversification, displaying a non-significant slope that is substantially weaker than that obtained for species (Fig. 5. Although subsampled Cenozoic genus diversity is generally higher than that of the Mesozoic, the distinction between Mesozoic and Cenozoic values is less prominent than that in the species-level analyses (Fig. 5). In particular, the genus diversities of Induan–Anisian (Early/Middle Triassic), Callovian/Oxfordian (Middle/Late Jurassic), and Campanian Asian tetrapods, and Campanian North American tetrapods are comparable to the range of values obtained for Cenozoic genera. This is consistent with the possibility that mammalian species, but not genera, are divided more finely by taxonomists than are dinosaurs, or other reptiles. However, we are cautious in accepting the results of our genus-level analyses. Genera may often be used as ‘form taxa’ to describe widespread dental morphologies, and some genera in the database [e.g. *Megalosaurus* [S1] represent ‘wastebasket taxa’, a problem that is less common for species than genera.

**References**

S1. Carrano MT, Benson RBJ, Sampson SD (2012) The phylogeny of Tetanurae (Dinosauria: Theropoda). J Syst Paleo 10: 211-300.
