# Supplementary figures and images for: Near-Stasis in the Long-Term Diversification of Mesozoic Tetrapods
Source: PLoS Biol. 2016 Jan 25;14(1):e1002359. doi: 10.1371/journal.pbio.1002359 (PMC4726655; doi:10.1371/journal.pbio.1002359)

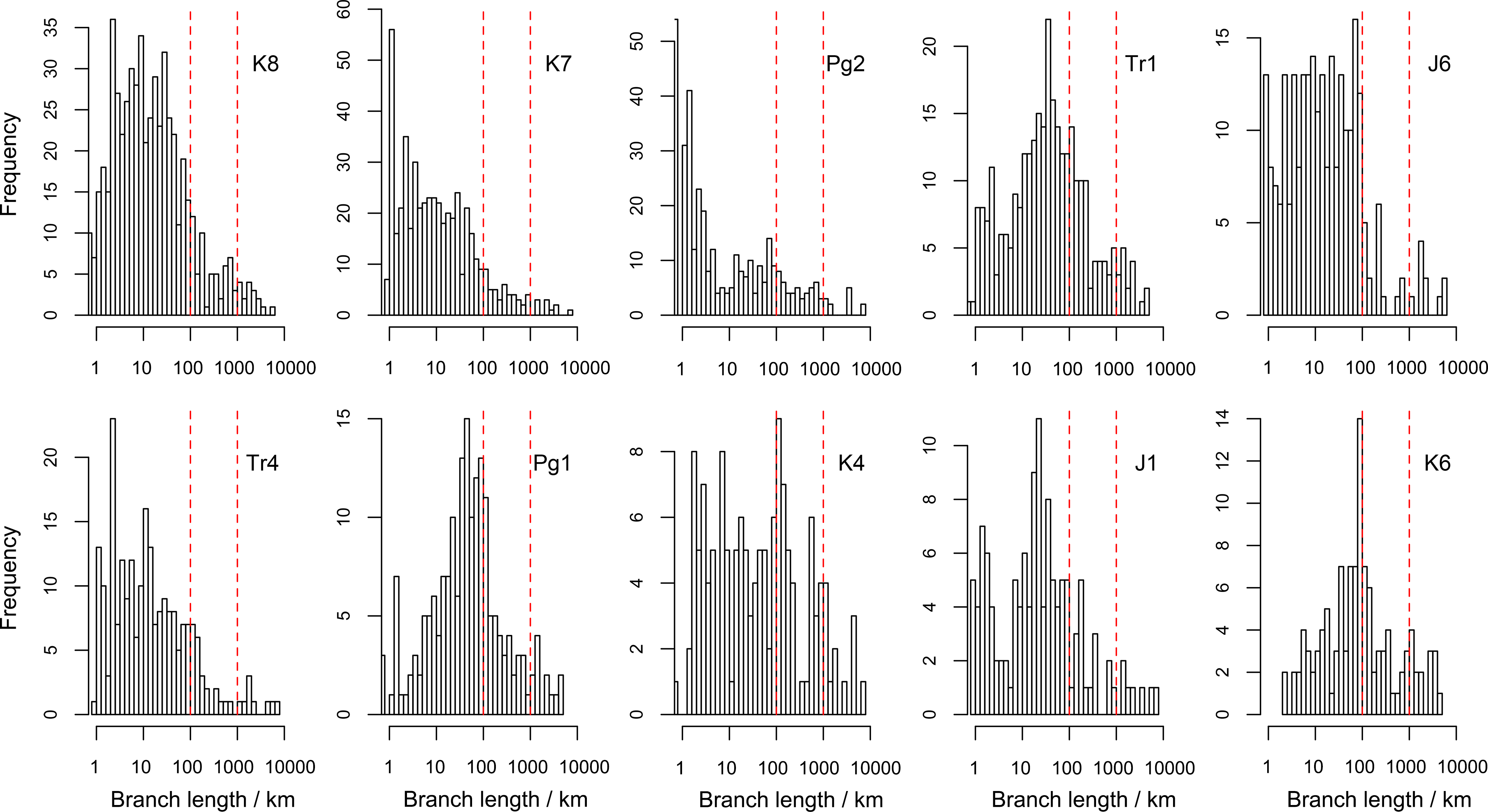

Supplement: S1 Fig — Interval name abbreviations are given in S1 Table. Red dashed lines indicate 100 km and 1,000 km. The data displayed in this figure can be accessed at http://doi.org/10.5061/dryad.9fr76 [90]. (TIF) [file pbio.1002359.s002.tif]

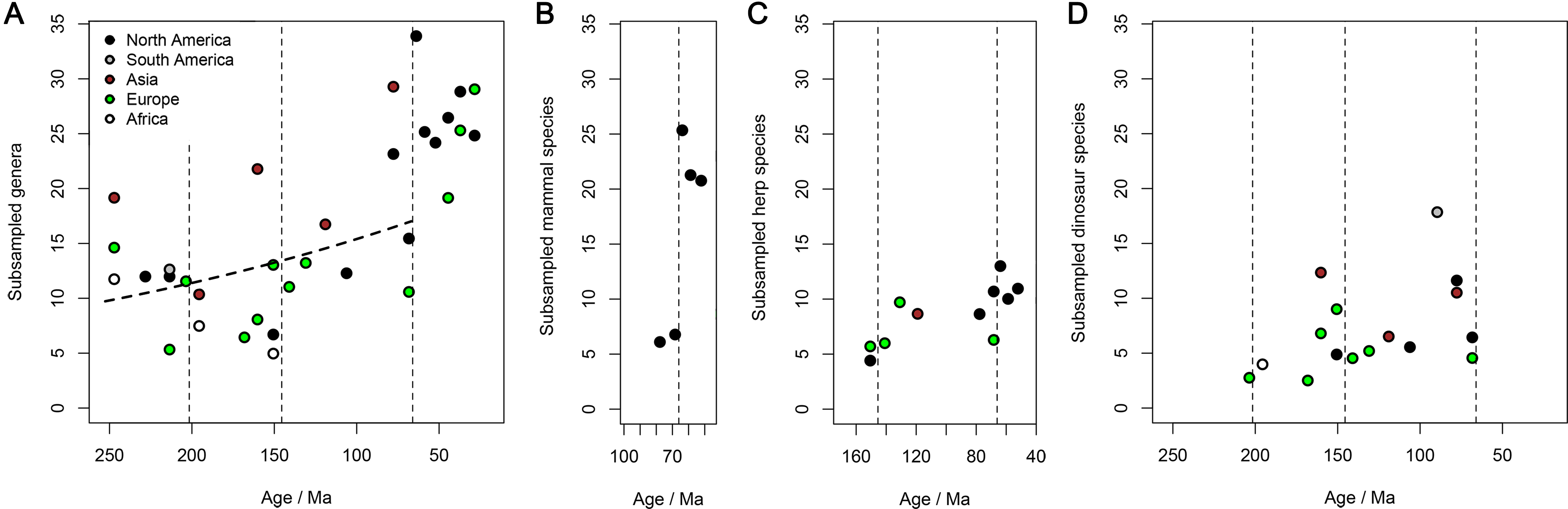

Supplement: S2 Fig — (A) Results for all tetrapods; the dashed line is the general linear model predicting subsampled regional genus diversity from geological age for the entire Mesozoic, modelling taxon counts as a Gaussian distribution and using a ln() link function (slope = -0.003; standard error of slope = 0.0015; p = 0.064; intercept = 3.03). (B–D) Results for mammals (B), non-mammalian, non-dinosaurian tetrapods (“herps”) (C), and dinosaurs (D). The data displayed in this figure can be accessed at http://doi.org/10.5061/dryad.9fr76 [90]. (TIF) [file pbio.1002359.s003.tif]
