# Supplementary material for: Near-Stasis in the Long-Term Diversification of Mesozoic Tetrapods
Source: PLoS Biol. 2016 Jan 25;14(1):e1002359. doi: 10.1371/journal.pbio.1002359 (PMC4726655; doi:10.1371/journal.pbio.1002359)
Supplement: S1 Table — Pg2 ends at 48.6 Ma. Occurrences were assigned to a time bin only if their stratigraphic age uncertainty was entirely contained within that bin. (DOCX) [file pbio.1002359.s004.docx]

| **Age of base** | **Abbreviation** |  | **Included stages** |
| --- | --- | --- | --- |
| 56.0 | Pg2 | Eocene | Ypresian |
| 61.6 | Pg1 | Paleocene | Selandian, Thanetian |
| 66.0 | Pg0 | Paleocene | Danian |
| 72.1 | K8 | Late Cretaceous | Maastrichtian |
| 83.6 | K7 | Late Cretaceous | Campanian |
| 93.9 | K6 | Late Cretaceous | Turonian, Coniacian, Santonian |
| 100.5 | K5 | Late Cretaceous | Cenomanian |
| 113.0 | K4 | Early Cretaceous | Albian |
| 126.3 | K3 | Early Cretaceous | Aptian |
| 133.9 | K2 | Early Cretaceous | Hauterivian, Barremian |
| 145.0 | K1 | Early Cretaceous | Berriasian, Valanginian |
| 157.3 | J6 | Late Jurassic | Kimmeridgian, Tithonian |
| 166.1 | J5 | Middle/Late Jurassic | Callovian, Oxfordian |
| 170.3 | J4 | Middle Jurassic | Bajocian, Bathonian |
| 182.7 | J3 | Early/Middle Jurassic | Toarcian, Aalenian |
| 190.8 | J2 | Early Jurassic | Pliensbachian |
| 201.3 | J1 | Early Jurassic | Hettangian, Sinemurian |
| 209.5 | Tr5 | Late Triassic | Rhaetian |
| 228.4 | Tr4 | Late Triassic | Norian |
| 237.0 | Tr3 | Late Triassic | Carnian |
| 241.5 | Tr2 | Middle Triassic | Ladinian |
| 252.2 | Tr1 | Early/Middle Triassic | Induan, Olenekian, Anisian |

**S1 Table. Composite 9 Myr time bins used in the present study.** Pg2 ends at 48.6 Ma. Occurrences were assigned to a time bin only if their stratigraphic age uncertainty was entirely contained within that bin. This table contains customised intervals that may differ from those at paleobiodb.org and fossilworks.org.
