# Supplementary material for: Near-Stasis in the Long-Term Diversification of Mesozoic Tetrapods
Source: PLoS Biol. 2016 Jan 25;14(1):e1002359. doi: 10.1371/journal.pbio.1002359 (PMC4726655; doi:10.1371/journal.pbio.1002359)
Supplement: S2 Table — (DOCX) [file pbio.1002359.s005.docx]

| **Region** | **Included countries** |
| --- | --- |
| North America | United States, Canada, Mexico. |
| South America | Argentina, Chile, Brazil, Bolivia, Colombia, Uruguay, Peru. |
| Asia | China, Mongolia, South Korea, Russian Federation, North Korea. |
| Europe | United Kingdom, France, Germany, Italy, Switzerland, Spain, Belgium, Germany, Romania, Sweden, Czech Republic, Denmark, Slovenia, Norway, Luxembourg, Netherlands, Ukraine, Hungary, Austria, Poland, Croatia, Portugal. |
| Africa | Zambia, Namibia, Zimbabwe, Mali, Angola, Ethiopia, Cameroon, Malawi, Senegal, Tanzania, Eritrea, Sudan, Kenya, Libya, Niger, Tunisia, Algeria, Lesotho, Morocco, South Africa. |

**S2 Table.** Countries included in our contiguous continental regions.
